# Comparison of Drainage Methods After Pyeloplasty in Children: A 14-Year Study

**DOI:** 10.3389/fped.2021.779614

**Published:** 2021-12-13

**Authors:** Xiangpan Kong, Zhenpeng Li, Mujie Li, Xing Liu, Dawei He

**Affiliations:** ^1^Department of Urology, Children's Hospital of Chongqing Medical University, Chongqing, China; ^2^Ministry of Education Key Laboratory of Child Development and Disorders, International Science and Technology Cooperation Base of Child Development and Critical Disorders, National Clinical Research Center for Child Health and Disorders, Chongqing Key Laboratory of Pediatrics, Chongqing Key Laboratory of Children Urogenital Development and Tissue Engineering, Chongqing, China

**Keywords:** pyeloplasty, hydronephrosis, ureteropelvic junction obstruction, drainage methods, outcomes

## Abstract

**Objective:** To summarize our experiences with drainage methods after laparoscopic pyeloplasty with a 14-year study.

**Methods:** We reviewed the data of the 838 children operated on for hydronephrosis due to congenital ureteropelvic junction obstruction (UPJO) between July 2007 and July 2020. Patients' demographics, perioperative details, postoperative drainage stents [including double-J stent, percutaneous trans-anastomotic (PU) stent, and trans-uretero-cystic external urethral stent (TEUS)], complications, hospital stay, and long-term follow-up outcomes were analyzed. Long-term follow-up was performed by outpatient visits and telephone follow-up. Moreover, we reviewed the details of nine cases of recurrence after laparoscopic pyeloplasty.

**Results:** Comparison of preoperative general data among the three groups indicated that there was no statistical difference in age, gender, and surgical side of the three groups. Statistical differences were found in the incidence of postoperative complications from the three postoperative drainage method groups, especially the incidence of reoperations (*p* < 0.01): there were six cases (3.19%) of recurrences in the TEUS group, two cases (0.36%) in the DJ group, and one case (0.93%) in the PU group. In the six recurrent cases from the TEUS group, four cases (44.4%) were found to have stenosis, and two cases (22.2%) have iatrogenic valvular formation.

**Conclusion:** Not all three types of drainage methods are suitable for drainage after pyeloplasty. Based on our findings, TEUS is not recommended.

## Introduction

Congenital ureteropelvic junction obstruction (UPJO) is one of the most commonly encountered abnormalities that are responsible for persistent hydronephrosis in children (1). The classic option of treatment for UPJO is pyeloplasty. Since the first descriptions of laparoscopic pyeloplasty (LP) in 1993 by Schlussel (2) and in 1995 by Peters (3), LP has become the gold standard in the treatment of UPJO, with its safety and minimal invasiveness. Usually, surgeons will choose to use a drainage stent after pyeloplasty; however, which drainage method is the best choice is still quite controversial (4, 5).

After LP, the choice of stent type has always been the focus of debate. For now, double-J (DJ) stent and percutaneous trans-anastomotic (PU) are widely used due to their reliable efficacy, but their disadvantages are also obvious, such as displacement and secondary anesthesia in the DJ stent (5–8) and urine leakage, kinks, and obstruction in the PU stent (5, 9, 10). Therefore, the ideal drainage method should be effective while being minimally invasive and safe. We used the trans-uretero-cystic external urethral stent (TEUS) approach to solve the problems caused by the DJ stent and PU stent; in the previous research (11), we proved it to be safe and effective by comparing it with the DJ stent, but there is a lack of verification of long-term follow-up results in the study.

After a long-term postoperative follow-up work, we found some abnormal results (postoperative recurrence rates were higher in children treated with TEUS than other drainage methods), which made us question the safety of this new drainage method. Therefore, we conducted this study to answer the question, summarize the relevant experience and findings, and share them with other scholars.

## Materials and Methods

### Patients and Data

We retrospectively reviewed 838 patients with congenital UPJO without other urinary system deformities between July 2007 and June 2020 in the Children's Hospital of Chongqing Medical University. All patients underwent standard LP according to Anderson–Hynes technique (12); surgeries were performed by three senior surgeons with extensive experience in pediatric urology surgery. Patients' demographics, data of preoperative and postoperative exams, perioperative details, complications, hospital stay, and regular postoperatively follow-up results were collected (the occurrence of long-term complications).

### Surgical Method and Follow Up

**Figure 1:**
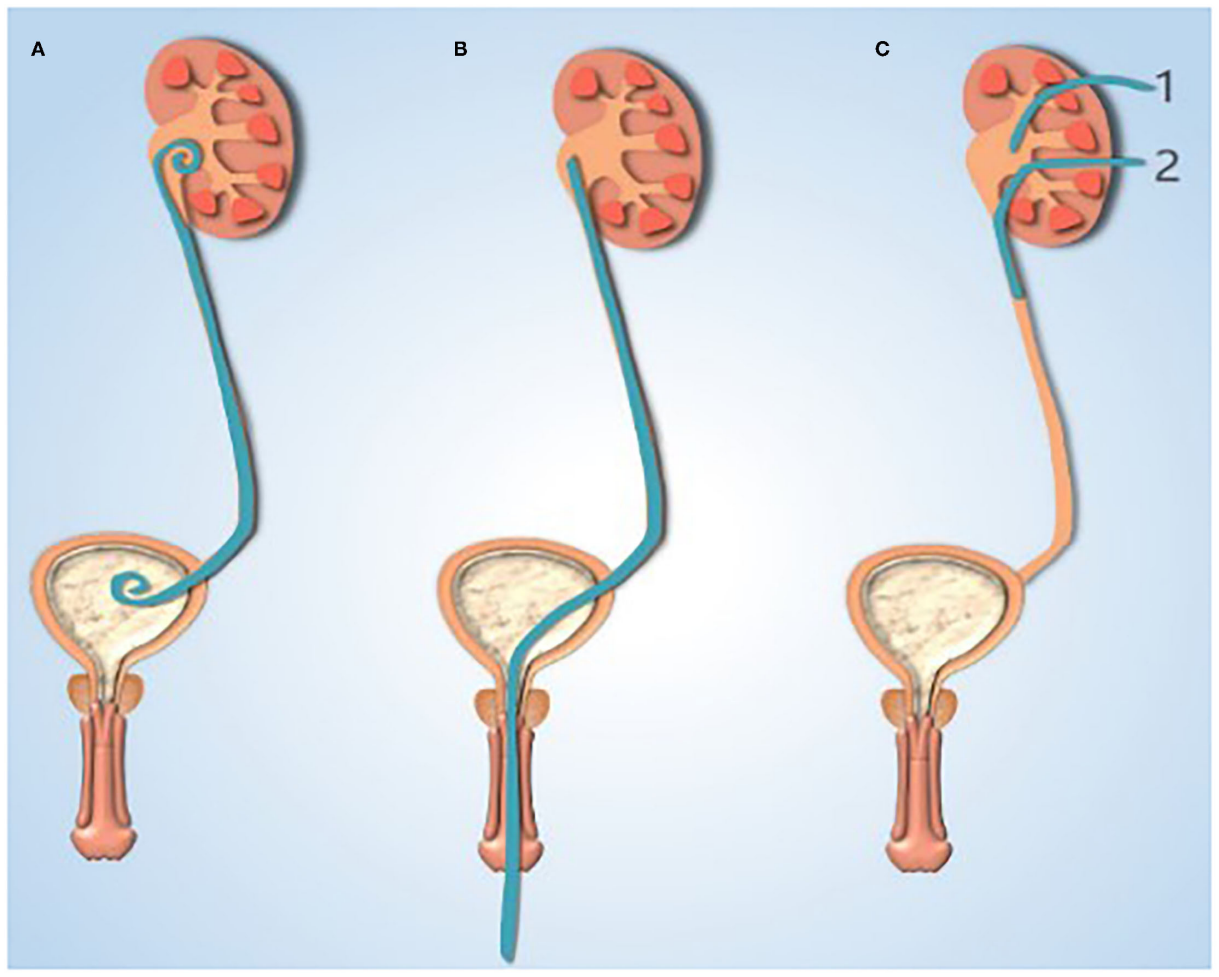
Schematic diagram of three types of postoperative drainage stents. **(A)** DJ stent. **(B)** TEUS. **(C)** PU stent: 1, drainage stent; 2, stent. DJ, double-J stent; TEUS, trans-uretero-cystic external urethral stent; PU, percutaneous trans-anastomotic.

Stenting is selected by the surgeon according to the preoperative or intraoperative situation. The TEUS stent was placed by a cystoscope preoperatively; a Fr3 or Fr4 stent was inserted in a retrograde fashion into the ureter via cystoscopy, with a Foley catheter placed in the bladder. The other stents were used intraoperatively (Figure 1). Seven to 10 days after surgery, the PU stent and TEUS stent were removed, while the DJ stent was removed about 1–4 weeks after surgery.

Follow-up included outpatient follow-up at 3 and 6 months and once a year after surgery. Patients who were followed up for <1 year or were lost to follow-up were excluded.

### Statistical Analysis

Postoperative complications were analyzed by the Clavien–Dindo system (13). Analyses were performed using SPSS^®^, version 25.0 (IBM Corp., Armonk, NY, USA). Qualitative or categorical variables were expressed as numbers and compared using the χ^2^ or Fisher’s exact test, as appropriate. Data were compared between groups using Students’ *t*-test or chi-square test. Data that did not comply with a normal distribution were expressed as median range and compared between groups using the Mann–Whitney test. All statistical tests were two-sided and performed with a significance level set at *p* < 0.05.

### Ethics Approval

We obtained ethical approval for this study from the local institutional research ethics board. Written informed consent for participation was signed by the guardian of the child when hospitalized.

## Results

This study included a total of 838 children who underwent LP. The demographics data (gender, age, and surgical side) of the three groups were not statistically significant (*p* > 0.05). From the comparison of the operative duration, intraoperative blood loss, and hospitalization duration of patients in the three groups, statistically significant differences were found between groups. The operative duration was significantly different between the DJ group and the other two groups (*p* < 0.05). Bleeding volume in the PU group was significantly different from that of the other two groups (*p* < 0.05). Hospitalization duration was statistically different among the three groups. Among them, compared with the other two groups, the DJ group had the shortest hospitalization duration and the shortest operation duration; the PU group had the most blood loss; and the TEUS group had the longest operation duration (Table 1).

**Table 1:** Patients' demographics and data of operation.

	**DJ group**	**TEUS group**	**PU group**	***p*-Value**
Number, *n*	543	188	107	-
Male, gender, *n* (%)	445 (82.0)	147 (78.2)	79 (73.8)	0.285
Age, months, median (IQR)	57 (14–91)	30 (11–83)	48 (13–83)	0.064
Side, left, n (%)	427 (78.6)	146 (77.7)	77 (72.0)	0.285
•Operative duration, min •Median (IQR)	100 (79–130)	120 (95–155)	115 (90–140)	0.000^*^
•Bleeding volume, ml •Median (IQR)	10 (5–10)	10 (5–10)	10 (5–15)	0.000^*^
•Hospitalization duration, days •Median (IQR)	12 (10–15)	15 (14–18)	18 (16–20)	0.000^*^

*DJ, double-J stent; TEUS, trans-uretero-cystic external urethral stent; PU, percutaneous trans-anastomotic; IQR, interquartile range*.

* *Significant*.

We calculated the time of the stent removal and postoperative complications in the three groups. The time of the stent removal of the three groups was 28.5 ± 12.2, 7.4 ± 1.8, and 10.9 ± 8.2 days, which was significantly different between groups. Meanwhile, the overall complication rate in the three groups was significantly different too. They are 24 (4.42%) cases, 23 (12.23%), and nine (8.41%) cases; especially, the incidence of reoperation in Group B (six cases) was significantly higher than in other groups (Table 2).

**Table 2:** The three drainage stents' removal time and complications.

	**DJ group (*n* = 543)**	**TEUS group (*n* = 188)**	**PU group (*n* = 107)**	***p*-Value**
Stent removal time, day (mean ± SD)	28.5 ± 12.2	7.4 ± 1.8	10.9 ± 8.2	0.000^*^
Complications, *n* (%)	24	23	9	0.001^*^
UTI (CDG II)	12 (50)	6 (26.1)	3 (33.3)	0.715
Urine leakage (CDG II)	10 (41.7)	6 (26.1)	4 (44.4)	0.299
Stent drop (CDG II)	0 (0)	3 (13.0)	1 (11.1)	-
Omental hernia (CDG II)	0 (0)	1 (4.35)	0 (0)	-
Paralytic intestinal obstruction (CDG IIIb)	0 (0)	1 (4.35)	0 (0)	-
Recurrence (CDG IIIb)	2 (8.3)	6 (26.1)	1 (11.1)	0.007^*^

*DJ, double-J stent; TEUS, trans-uretero-cystic external urethral stent; PU, percutaneous trans-anastomotic; UTI, urinary tract infection; CDG, clavien-dindo grading*.

* *Significant*.

At last, we collected clinical data from the nine children (six boys and three girls) who underwent reoperation; all developed severe hydronephrosis before the first surgery. After the first operation, five children had a recent complication (two cases of urinary tract infection (UTI), two cases of anastomotic obstruction, and one case of persistent hematuria). In the choice of postoperative drainage stent, we used TEUS in six children, the DJ stent in two children, and the PU stent in one child. During the reoperation, surprisingly, four cases showed that the ureteropelvic junction still had scar stenosis, and two cases showed iatrogenic valve; it is worth noting that TEUS was used in all these six children. In the remaining three reoperation cases, two cases were found to have surrounding tissues adhering to the stent, ureteropelvic junction did not have obvious stenosis, and these patients had UTI after the first surgery. The last case had angulation distortion.

## Discussion

More than 30 years ago, open pyeloplasty (OP) was the gold standard for the treatment of UPJO. The first LP was reported in 1993 (2), which is safe, reliable, and minimally invasive. LP has gradually become the standard method for the treatment of UPJO in children. However, due to the peculiarities of children, which type of drainage method is the best choice has been controversial after pyeloplasty.

Should a stent be used after LP, and if a stent is used, which stent is the most ideal?

At present, there are two kinds of stent tubes widely used: the DJ stent and PU stent. Recently, Sarhan et al. (5) reported a multicenter study of the efficacy of drainage methods in 175 children between the two groups, which showed no significant difference in the incidence of postoperative complications or long-term outcomes. DJ stent insertion provides a shorter hospital stay, but a second operating room visit and anesthesia for removal are unavoidable. Similarly, in the study of Irene et al. (8), they also compared the costs incurred by the two drainage methods, and they believed that the DJ and PU stents were equivalent in terms of overall complications and success rate. Moreover, PU stents can avoid the need for additional general anesthesia and reduce overall hospital costs. Therefore, the advantages of the DJ stent are that is minimally invasive, safe, and reliable, but it requires reoperation to remove the stent. The PU stent has the advantages of convenient stent removal and precise curative effect and the disadvantages of more trauma.

Since some catheter-related complications are inevitable with all types of drainage methods, what is the efficacy of stent-less pyeloplasty? Bayne et al. (14) proved that the incidence of postoperative urinary leakage was significantly higher in the stent-free group than in the stent-less group in their study. And in another meta-analysis reported by Liu (9) to evaluate the efficacy and safety of the DJ stent, PU stent, and stent-less pyeloplasty in pediatric pyeloplasty, the network meta-analysis (NMA) results showed that there were no significant differences between the three groups in surgical duration, surgical success rate, length of hospital stay, improvement in renal function, overall complications, and recurrence rates. Compared with the stent-less group, the incidence of postoperative pain was higher for the DJ stent and PU stent. The urine leakage rate of the DJ stent was lower than that of the PU stent and stent-less pyeloplasty. No significant differences were observed in other types of complications such as UTI, stent displacement, and postoperative recurrence. This is consistent with other similar studies (15, 16), so the cost of stent-less pyeloplasty is an unavoidable high incidence of urinary leakage. Unfortunately, almost all postoperative urine leakage needs to be treated by intubation; it means that reoperation is conducted within a short period of time after the first surgery, which is unacceptable for children and their parents, and it may cause doctor–patient conflict and bring great challenges to clinical work.

Combined with the above discussions, we find that stent-less pyeloplasty is the most minimally invasive, but it has a high incidence of urinary leakage. Combined with the results of the other studies (5, 6, 10, 15–17), we found that the advantages of the DJ stent are that it is safe, reliable, effective, and more minimally invasive, while the removal time of the PU stent is shorter, which can reduce the occurrence of catheter-related complications. And the disadvantages are obvious too, such as issues with anesthesia during DJ stent removal and the high risk of urine leakage associated with the PU stent. In order to solve these problems, we tried a new drainage stent, TEUS. This drainage stent through the natural cavity solves not only the problem of DJ stent removal difficulty but also the problem of PU stent urine leakage around the catheter. Is this drainage method safe and effective? In an early short-term retrospective study, we compared the efficacy of the TEUS stent and DJ stent, and we found that in addition to the operation duration of the TEUS group, which was longer than that of the DJ group (*p* < 0.05), there was no difference in intraoperative blood loss, length of hospital stay, and incidence of complications [10 cases (22.2%) and eight cases (20%) of catheter-related complications in the DJ group and TEUS group, respectively (*p* > 0.05)] (*p* > 0.05). However, this study on the safety of TEUS lacked long-term follow-up results.

With the increased time of follow-up, we compared the removal time of stents and incidence/types of postoperative complications of 884 patients in the LP group who respectively used the DJ stent, TEUS, and PU stent for drainage. One unexpected finding was the extent to which the removal time of stents and overall complication rate of the three groups were statistically different, and the average catheter duration of the three groups was as follows: DJ group (28.5 days), TEUS group (7.4 days), and PU group (10.9 days). The incidence of postoperative complications in the three groups was as follows: 24 cases (4.42%) in the DJ group, 23 cases (12.23%) in the TEUS group, and nine cases (8.41%) in the PU group; especially, the incidence of reoperation in the TEUS group (six cases, 26.1%) was significantly higher than that in the other groups (two cases, 8.3%; one case, 1.1%). The finding that the incidence of postoperative complications was significantly different among the three groups was seriously inconsistent with the previous conclusion. Then what causes the postoperative recurrence rate of the TEUS group to be significantly higher than that of the other groups?

Current studies suggest that stenting and drainage after pyeloplasty are necessary to facilitate anastomotic healing and reduce urinary leakage (18). Both the DJ and PU stents have this function, but the TEUS stent had no supporting effect due to its special structure. In addition, TEUS is placed prior to pyeloplasty, which means that the renal pelvis will be emptied before pyeloplasty begins, and it may affect the judgment of the length of the stenosis, which may lead to residual stenosis. Moreover, the TEUS was inserted before surgery, which interferes with the surgical field during surgery, which is also not conducive to complete resection and suture of the stenosis and may eventually lead to residual stenosis and inaccurate suture. These hypotheses were also confirmed by pathological findings during reoperation (four cases with residual stenosis and two cases with close adhesion to surrounding tissues). And to further test this hypothesis, we are now conducting further experimental studies. Now, we do not recommend the use of TEUS stents, and we suggest that other scholars should not ignore our findings when trying new stents.

Compared with other reported literature (5, 8, 13–16), the advantages of our study lie in the long follow-up time and importantly in the number of patients. To our knowledge, this is the first long-term follow-up of TEUS and study of the results. The limitations of this study are that the data were retrospectively analyzed, the study group was not randomized, and the study was a single-center observation.

In summary, not all three types of drainage methods are suitable for pyeloplasty. We suggest that the use of the TEUS stent should be performed carefully, and we suggest that other scholars should not ignore our findings when trying new stents.

## Data Availability Statement

The raw data supporting the conclusions of this article will be made available by the authors, without undue reservation.

## Ethics Statement

Written informed consent was obtained from the individual(s), and minor(s)' legal guardian/next of kin, for the publication of any potentially identifiable images or data included in this article.

## Author Contributions

XL and DH contributed to conception and design. DH contributed to administrative support. XK, ZL, and ML contributed to collection and assembly of data. XK contributed to manuscript writing. All authors contributed to manuscript revision, read, and approved the submitted version.

## Funding

This study was funded by the Special Key Project of Chongqing Technology Innovation and Application Development (No. Cstc2019jscx-tjsbX0003).

## Conflict of Interest

The authors declare that the research was conducted in the absence of any commercial or financial relationships that could be construed as a potential conflict of interest.

## Publisher's Note

All claims expressed in this article are solely those of the authors and do not necessarily represent those of their affiliated organizations, or those of the publisher, the editors and the reviewers. Any product that may be evaluated in this article, or claim that may be made by its manufacturer, is not guaranteed or endorsed by the publisher.

## Abbreviations

UPJO: ureteropelvic junction obstruction
LP: laparoscopic pyeloplasty
DJ: double-J
PU: percutaneous trans-anastomotic
TEUS: trans-uretero-cystic external urethral stent.
